# Environmentally persistent free radicals lead to selective inhibition of CYP1 monooxygenase activities, and increased production of reactive oxygen species by reaction uncoupling

**DOI:** 10.3389/fpubh.2025.1531134

**Published:** 2025-06-10

**Authors:** J. Patrick Connick, Amari A. Stepter, George F. Cawley, Marilyn K. Eyer, Wayne L. Backes

**Affiliations:** Department of Pharmacology and Experimental Therapeutics, and The Stanley S. Scott Cancer Center, Louisiana State University Health Science Center, New Orleans, LA, United States

**Keywords:** environmentally persistent free radicals, cytochrome P450, CYP1A1, CYP1A2, CYP1B1, reactive oxygen species, enzyme inhibition, protein-protein interactions

## Abstract

This study focuses on the effect of Environmentally Persistent Free Radicals (EPFRs) on the P450 enzymes of the CYP1 family. EPFRs are a component of particulate pollutants, that are stable in the environment, but can generate free radicals, leading to oxidative stress and subsequent toxicity of the respiratory, cardiovascular, and immune systems once they enter an organism. The results show differences in the ability of EPFRs to inhibit CYP1-dependent substrate metabolism, with CYP1B1 being inhibited to the greatest extent. There also were differences in the ability of EPFRs to disrupt the POR•CYP1 complex, with CYP1B1 being the only form where EPFRs disrupted POR•CYP1B1 complex formation. Despite the inhibition of substrate metabolism, each CYP1 enzyme, when reconstituted with NADPH-cytochrome P450 reductase (POR) was able to synergistically stimulate the generation of reactive oxygen (ROS) in the presence of particulate matter. Interestingly, both POR and the CYP1 enzymes were able to stimulate ROS generation, even when in partial reconstituted systems where only one of the proteins was present. However, when both POR and CYP1 were combined in a complete reconstituted system, ROS generation was synergistically stimulated.

## Introduction

Environmentally persistent free radicals (EPFRs) represent a newly observed class of pollutants that are formed by incomplete combustion or thermal treatment of organic matter in the presence of metals and are found at Superfund sites as well as other locations (1–5). EPFRs have been shown to be stable in the environment, having half-lives of several days or longer (6, 7). However, once they enter an organism, they can rapidly generate free radicals, leading to oxidative stress and subsequent toxicity of the respiratory, cardiovascular, and immune systems (3, 7–15). Based on previous work, the majority of these EPFR effects were mediated through activation of the aryl hydrocarbon receptor (AhR), which can occur through multiple mechanisms (16–18). One of the major downstream effects of AhR activation is the induction of P450 enzymes that belong to the CYP1 family, which consists of CYP1A1, CYP1A2, and CYP1B1 (16, 17).

Cytochromes P450 are enzymes responsible for the oxidation of a wide variety of both exogenous and endogenous substrates, with several forms being capable of uncoupling, i.e., generating ROS instead of metabolizing substrate (19, 20). CYP1 enzymes are known to uncouple in addition to metabolizing numerous carcinogenic substances to their bioactive forms (21–23). As induction of CYP1 expression is a major downstream effect of Ah receptor activation, we became interested in examining the mechanisms governing this response.

In addition to EPFR-mediated activation of AhR, our laboratory showed that EPFRs caused generalized inhibition of multiple P450 enzymes (12, 13, 24, 25). Our results demonstrated that EPFRs dramatically inhibit CYP1A1, CYP1A2, CYP2B, CYP2E1, CYP2D2, and CYP3A from rat liver microsomes. Similar results were obtained with rabbit CYP1A2 and CYP2B4 (12, 13, 24).

Consequently, there are several reasons for interest in how CYP1 proteins respond to EPFR exposure. EPFRs are known to produce an Ah receptor response. Potentially, AhR activation is mediated by EPFR-mediated generation of ROS, which can lead to the metabolism of tryptophan to 6-formyindolo[3,2-b]carbazole (FICZ) (26–28). Under normal conditions, FICZ is inactivated by CYP1 enzymes, which leads to the termination of AhR activation. However, if CYP1 activities are inhibited in the presence of EPFRs, this may inhibit the inactivation of the endogenous agonists (e.g., FICZ) and prolong the response.

The goal of this study was to compare the effects of EPFRs on the activities of each of the CYP1 enzymes. Although previous studies suggested that this inhibition was the result of disruption of the physical complex between POR and CYP1A (24), BRET studies showed that complex formation between POR•CYP1A1 and POR•CYP1A2 was not affected by EPFRs. In contrast, the POR•CYP1B1 complex did appear to be disrupted by the presence of EPFRs. In general, monooxygenase activities for each of the enzymes were inhibited by EPFR treatment; however, in each of these cases, non-EPFR particulates also showed an inhibitory response. Despite the inhibition of monooxygenase activities, EPFR exposure led to an increase in the formation of reactive oxygen species (ROS) when a CYP1-containing reconstituted system was present. Interestingly, a functional reconstituted system was not required for EPFR-mediated ROS stimulation, where either POR or the CYP1s alone could stimulate this oxidative response. Overall, the results show that despite the sequence similarities among these closely related enzymes, each form has unique characteristics with regard to their response to EPFR exposure.

## Materials and methods

### Materials

All chemicals and reagents were of the highest quality commercially available. Dilauroylphosphatidylcholine (DLPC), NADPH, magnesium chloride, phenylmethylsulfonyl fluoride, catalase, calcium chloride, and NaCl were purchased from Sigma (St. Louis, MO). Sodium bicarbonate, potassium chloride, and monobasic potassium phosphate were obtained from Mallinckrodt Pharmaceuticals (Hazelwood, MO). Dibasic potassium phosphate was obtained from EMD chemicals (Gibbstown, NJ).

The expression system for human CYP1A1 was a gift from Dr. Doug-Young Ryu (Seoul National University, South Korea). Human CYP1A2 was a gift from Dr. Fred Guengerich (Vanderbilt University), and CYP1B1 was cloned from the cDNA that was purchased from LSBio (Shirley, MA). The expression system for NADPH-cytochrome P450 reductase was obtained from Dr. Lucy Waskell (U. Michigan, Ann Arbor).

Dulbecco's modified Eagle's medium (DMEM), NuPAGE 10% Bis-Tris gel, Lipofectamine 2000, PBS, EDTA, bis(sulfosuccinimidyl)suberate (BS3), and SDS were purchased from Invitrogen (Eugene, OR). HEK293T/17 cells were obtained from ATCC (Manassas, VA). Glycine and HEPES were obtained from Biomatik Corporation (Ontario, Canada). Coelenterazine 400a, coelenterazine h, kanamycin, and DTT were purchased from BioSignal Packard (Waltham, WA). Zeocin was obtained from InvivoGen (San Diego, CA). 7-Benzyloxyresorufin (7BR) was purchased from Anaspec (Fremont, CA).

The human P450s and POR used for BRET were wild-type proteins without any sequence modifications. The cDNA for CYP1B1 was purchased from LSBio (Shirley, MA). The cDNAs for CYP1A1, CYP1A2, and POR were obtained from GE Healthcare/Dharmacon (Lafayette, CO).

### Generation of EPFR and non-EPFR particles

CAB-O-SIL EH-5 was purchased from Cabot Corporation (Billerica, MA). This fumed silica was impregnated with 5% (w/w) copper oxide to generate the non-EPFR particle, CuO-Si, as described previously (5, 12, 13). Silica was impregnated with copper nitrate hemipentahydrate by incubation in a 0.1 M solution for 24 h at room temperature. Subsequently, the silica was dried at 120°C for 12 h and then heated for 5 h in air at 450°C. To generate the EPFR (MCP230), the particles were then placed in vacuum (<10^−2^ torr) and heated to 230°C before being exposed to 2-chlorophenol vapors at 10 torr in a custom-made vacuum exposure chamber for 5 min. The samples were cooled to room temperature and evacuated for 1 h (10^−2^ torr). The radical contents of the EPFRs were analyzed by electron paramagnetic resonance (EPR) spectroscopy as described previously (6) and had spin contents greater than 1 × 10^17^ spins/g.

### Preparation of reconstituted systems

Reconstituted systems were prepared as described in previous reports (12, 13, 24). Briefly, a stock suspension of DLPC (8 mM in 0.05 M HEPES containing 100 mM NaCl, 20% glycerol, and 0.1 mM EDTA, pH 7.5) was bath sonicated until clarification. DLPC, POR, and the CYP1 enzymes were mixed at 160:1 (DLPC:P450) and incubated for 2 h prior to assay.

### 7-benzyloxyresorufin-o-dealkylase assay

CYP1 activities were estimated by monitoring the conversion of the substrate 7-benzyloxyresorufin to resorufin. The final concentrations of the assay components were: reconstituted system ([DLPC]:[CYP1] (160:1) and POR as indicated), 7BR (32 μM), in 50 mM HEPES, pH 7.5, containing 15 mM magnesium chloride and 0.1 mM EDTA (29–31). Results are expressed as the mean ± SD for at least 3 determinations.

### Measurement of complex formation by bioluminescence resonance energy transfer (BRET)

In an effort to determine if the redox complexes between POR and the different CYP1 proteins were disrupted by EPFR exposure, we used BRET to measure physical complex formation. HEK293T/17 cells were co-transfected with a *Renilla* luciferase-labeled POR (POR-Rluc) vector and GFP-labeled CYP1 (CYP1-GFP) vectors. Transfections were performed to induce expression of the tagged proteins at a high (>10×) CYP2E1-GFP:POR-Rluc ratio to ensure that BRET signals generated were near BRET_max_ and were not sensitive to small changes in expression ratio. Assays were performed as described previously with the addition of a 30-min incubation step after the addition of MCP230 or CuO-Si particles at a range of concentrations (32). Controls included untransfected cells, cells expressing a GFP-Rluc fusion protein, and cells expressing only POR-Rluc to normalize the BRET signal at each MCP230 or CuO-Si concentration.

### Measurement of ROS generation (DCF metabolism)

Metabolism of dichlorofluorescin (DCFH) was used to assess ROS generation with the CYP1-containing reconstituted systems, using a modification of previous methods (33–38). As DCF generation from the available substrate dichlorofluorescein diacetate (DCFH-DA) first requires its conversion to DCFH by cellular esterases (34, 35, 39), we generated DCFH by preincubation of DCFH-DA with porcine esterase prior to addition of NADPH. In preliminary experiments, the optimal time for generation of DCFH by porcine esterase (at 40 units/ml) was shown to be 3 min.

The reaction mixture contained 8 μM DCFH-DA, 40 units/ml of porcine esterase, either CuO-Si or MCP230 in 40 mM Tris pH 7.4, and the reconstituted systems containing POR and CYP1's. Experiments were run using 96-well plates under conditions where buffer composition and access to light were controlled. Samples were preincubated at 37°C for 3 min to generate DCFH, followed by the addition of 500 μM NADPH. The rate of increase in fluorescence was measured using excitation and emission wavelengths of 488 nm and 535 nm, respectively.

### Statistical analysis

Data are expressed as the mean ± SD in each of the figures, with the number of determinations described in the figure legends. In studies examining ROS generation, the slopes of the lines were calculated using a linear regression analysis, and significance was determined using an F test. Significance was determined using the Bonferroni *post hoc* test.

## Results

**Figure 1:**
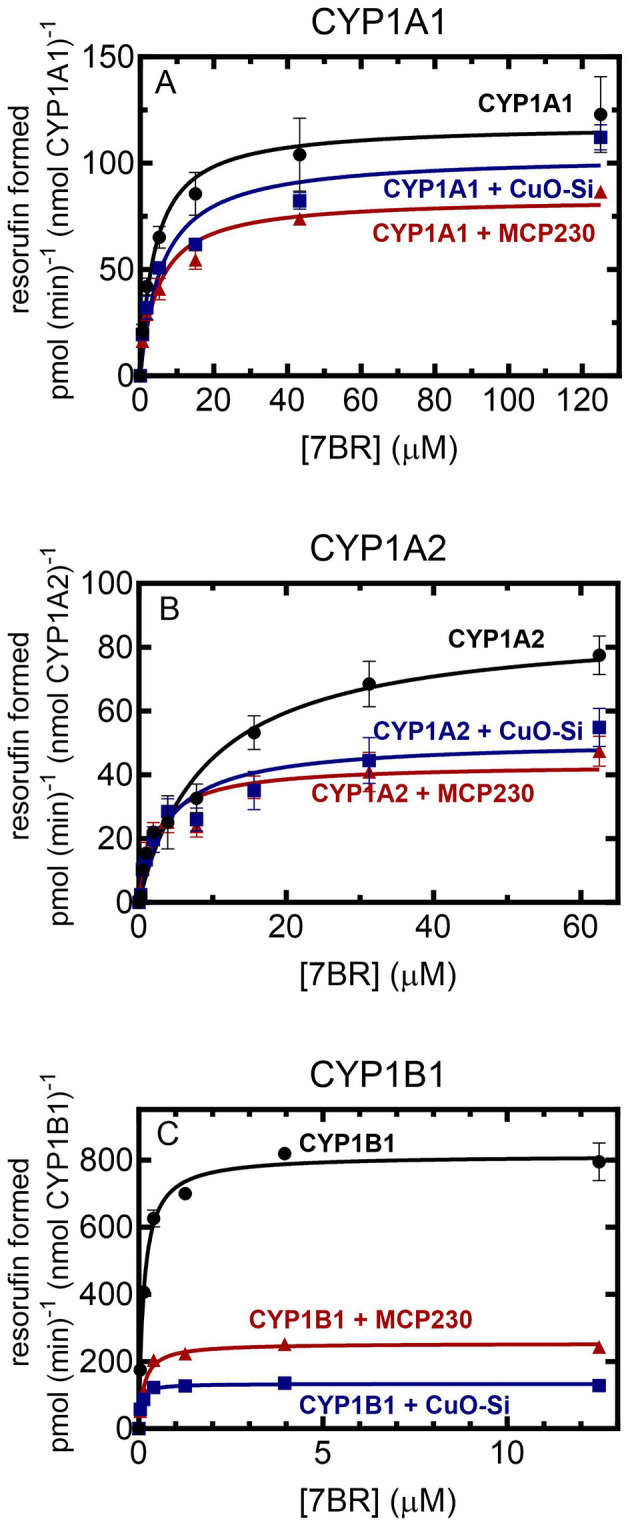
Effect of EPFR and non-EPFR particles on CYP1-mediated 7-benzyloxyresorufin metabolism as a function of substrate concentration. 7BR metabolism was examined in reconstituted systems containing 0.02 μM CYP1 and 0.2 μM POR in a reconstituted system in DLPC (160:1 DLPC:CYP1) as a function of substrate concentration in the presence and absence of 0.15 mg/ml non-EPFR (CuO-Si) and EPFR (MCP230) particles. The data points represent the mean ± SD for 3 determinations. **(A)** CYP1A1; **(B)** CYP1A2; and **(C)** CYP1B1.

In a previous report, we showed that EPFRs inhibited rat (12) and rabbit CYP1A2-mediated monooxygenase activity (24). Therefore, the goal of the study was to examine the effect of EPFR and non-EPFR particles on human CYP1s. As a first step, the effect of the addition of these particles on 7-benzyloxyresorufin metabolism was examined as a function of substrate concentration (Figure 1). Although all the CYP1s were able to metabolize 7BR, the kinetics of the responses were quite different. First, in the absence of substrate, the CYP1s exhibited different kinetic responses. The maximal activities of CYP1A1 and CYP1A2 were similar (118 and 87 pmol (min)^−1^ (nmol P450)^−1^ (Figures 1A, B). In contrast, CYP1B1 was a much more effective catalyst (Figure 1C), having a V_max_ about 8-fold higher (810 pmol (min)^−1^ (nmol P450)^−1^. A similar result was observed with the K_m_ values for the substrate 7-benzyloxyresorufin. CYP1A1 and CYP1A2 had similar K_m_ values (4.0 μM and 9.4 μM, respectively), whereas the K_m_ for CYP1B1 was about 30-fold lower (0.13 μM).

### Effect of EPFR and non-EPFR particles on activities as a function of substrate concentration

Each of the P450s was unique with regard to the effect of the particles on activities. In general, both the EPFR (MCP230) and the non-EPFR particle (CuO-Si) showed similar degrees of inhibition of 7-benzyloxyresorufin-O-dealkylation (BROD); however, there were differences when comparing the different CYP1s. At saturating substrate and POR, CYP1A1 activities were only modestly inhibited by particle exposure (Figure 1A). In the presence of the non-EPFR particle, there was a trend toward a lower V_max_ and higher K_m_, but these changes were not significant (Table 1). However, the EPFR did cause a significant decrease in V_max_.

**Table 1:** Effect of EPFR and non-EPFR particles on the kinetic constants for the CYP1s as a function of 7-benzyloxyresorufin concentration.

	**No particles**		**CuO-Si**		**MCP230**	
	**V**_max_ **pmol(min)**^−1^**(nmol P450)**^−1^	**K**_m_ μ**M**	**V**_max_ **pmol(min)**^−1^**(nmol P450)**^−1^	**K**_m_ μ**M**	**V**_max_ **pmol(min)**^−1^**(nmol P450)**^−1^	**K**_m_ μ**M**
CYP1A1	118 ± 5.0	4.00 ± 0.72	103 ± 5	5.97 ± 1.21	83.5 ± 3.1^*^	4.92 ± 0.76
CYP1A2	87.4 ± 4.5	9.37 ± 1.44	50.7 ± 2.8^*^	3.88 ± 0.80^*^	43.3 ± 2.1^*^	2.45 ± 0.48^*^
CYP1B1	814 ± 12	0.13 ± 0.10	134 ± 2^*^	0.06 ± 0.01^*^	254 ± 4^*^,	0.14 ± 0.01

Data were calculated from the Michaelis-Menten curves shown in Figure 1, and are expressed as the mean ± SE. Significant differences were indicated based on values outside the 95% confidence intervals.

* Significantly different (*p* < 0.05) from “no particles”.

y Significantly different (*p* < 0.05) from the CuO-Si group.

Both types of particles produced a greater degree of inhibition of CYP1A2-mediated BROD than seen with CYP1A1 (Figure 1B). In each case, the V_max_ decreased in the presence of either CuO-Si or MCP230. Interestingly, the K_m_ values actually decreased after exposure to either particle species (Table 1). These results indicate that both particles are equally effective inhibitors of CYP1A2-mediated BROD. Furthermore, the decrease in K_m_ values suggests that inhibition will not be observed at subsaturating substrate, at least for 7-benzyloxyresorufin.

CYP1B1 was inhibited to a much greater extent by both particle species when compared to the CYP1A enzymes (Figure 1C). The V_max_ was dramatically decreased by both particles, with the non-EPFR particle being inhibited to a greater extent than MCP230. Despite the already high affinity (very low K_m_) of CYP1B1 for 7-benzyloxyresorufin, the K_m_ for the non-EPFR (CuO-Si) was further decreased (Table 1). Taken together, these results may suggest that the non-EPFR particle will not be as inhibitory at very low substrate concentrations.

### Effect of EPFRs on the complex between POR and CYP1s

Several of our previous reports have shown that the EPFR, MCP230, was a stronger inhibitor of several rodent P450 enzymes than the non-EPFR particle, CuO-Si, particularly at subsaturating NADPH-cytochrome P450 reductase concentrations (12, 13, 24, 25). As previous data suggested that EPFRs may be inhibiting P450 function by disruption of the POR•P450 complex, we examined the potential of EPFR and non-EPFR particles to inhibit the human CYP1s by disrupting the formation of the POR•CYP1 complex using bioluminescence resonance energy transfer.

**Figure 2:**
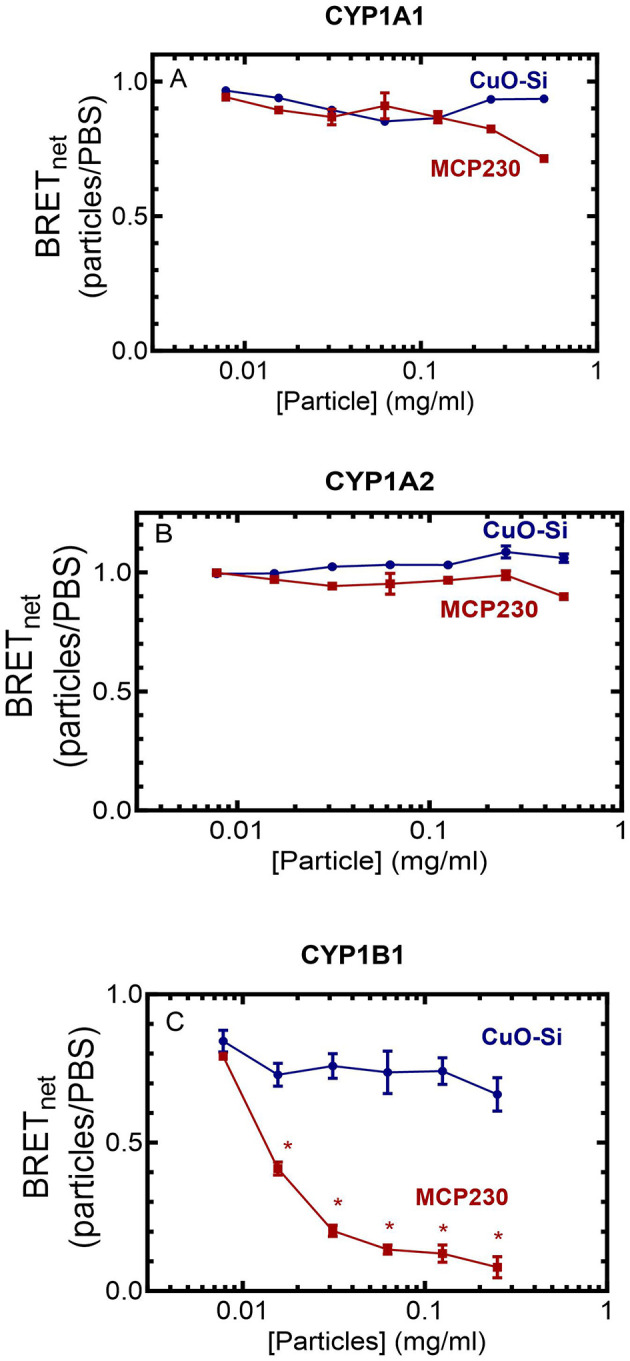
Effect of EPFR and non-EPFR particles on the POR•CYP1 redox complexes. HEK 293T/17 cells were transfected with plasmids coding for POR-Rluc and each of the CYP1-GFP constructs. After 24 h, complex formation between POR and the CYP1 enzymes was examined using BRET at a range of particle concentrations. Data points represent the mean ± the SD for triplicate determinations from a single group of cells. Previous experiments were conducted with adjustments to transfection conditions for optimization of protein expression, generating similar results (not shown). **(A)** CYP1A1, **(B)** CYP1A2, and **(C)** CYP1B1. ^*^p < 0.05.

Figure 2 shows that the different P450s have very different responses to the presence of EPFRs. Neither the EPFR nor the non-EPFR particles were capable of disrupting the physical POR•P450 complexes for CYP1A1 or CYP1A2 (Figures 2A, B). However, a different response was observed for CYP1B1, where the POR•CYP1B1 complex was significantly inhibited by MCP230 at concentrations as low as 0.03 mg/ml MCP230 (Figure 2C). This is in contrast to the non-EPFR CuO-Si, where POR•CYP1B1 complex formation was not affected. These results support the premise that despite their sequence similarities, the functional characteristics of the CYP1 enzymes differed dramatically.

### Effect of EPFRs on CYP1-mediated ROS production

Each of the CYP1 enzymes is known to uncouple product formation from NADPH consumption, leading to the generation of reactive oxygen species. In light of the significant inhibition of monooxygenase formation by MCP230 (as well as the non-EPFR CuO-Si), the ability of these particle species to affect ROS production was examined using reconstituted systems containing POR and the different CYP1 enzymes in the lipid DLPC similar to previous studies with CYP2E1 (25). ROS formation was monitored using DCFH-DA, a substrate that is normally converted to DCFH by cellular esterases. As we are using a defined reconstituted system that does not contain esterases, we converted DCFH-DA to DCFH with a 3-min preincubation with porcine esterase. After preincubation of the DCFH-DA and the reconstituted system, NADPH was added and formation of the fluorescent product DCF was used to measure ROS generation.

**Figure 3:**
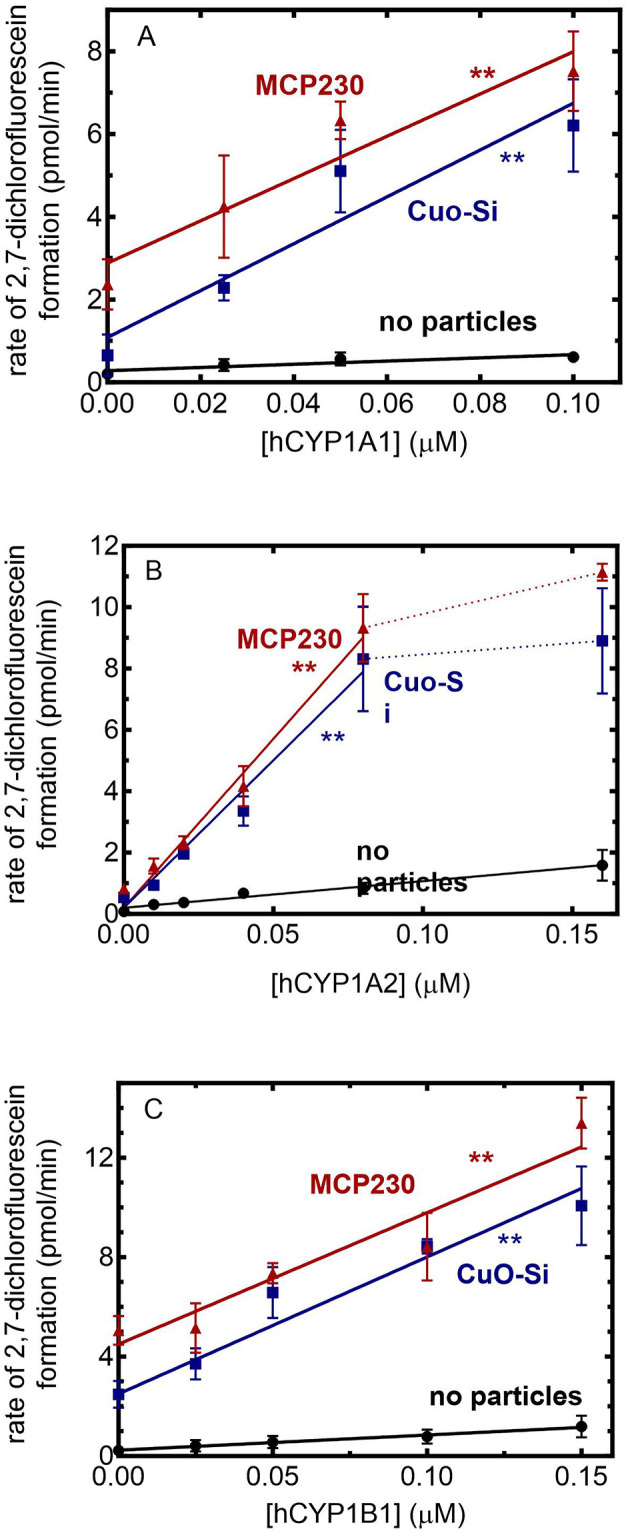
Effect of EPFRs on CYP1-mediated ROS formation. ROS generation was monitored using the formation of the fluorescent product DCF in the presence of 0.1 mg/ml of particles as a function of the POR/CYP1 reconstituted system. The POR:CYP1 ratio was 2:1. The results are the mean ± SD for at least 3 determinations. **(A)** CYP1A1: The slopes of the lines were 3.9, 56.6, and 51.1 (min)^−1^(μM CYP1A1)^−1^. Both treatment groups were determined to be significantly different from the no particle group (*p* < 0.01). **(B)** CYP1A2: The slopes of the lines, taken over the linear response range were 11.1, 67.2, and 79.6 (min)^−1^(μM CYP1A2)^−1^. Both treatment groups were determined to be significantly different from the no particle group (*p* < 0.01). **(C)** CYP1B1. The slopes of the lines were 6.1, 55.1, and 52.9 (min)^−1^(μM CYP1B1)^−1^. Both treatment groups were determined to be significantly different from the no particle group ^**^(*p* < 0.01).

Using this method, the ability of the CYP1 enzymes to generate ROS in the presence of CuO-Si and MCP230 was measured. Each of the CYP1 enzymes led to an increase in ROS generation when both EPFR and non-EPFR particles were present. When comparing each of the P450 enzymes, differences were observed in the ability of the P450s alone to generate ROS (Figures 3A–C, black lines). Based on the slopes of the lines in the absence of particles, CYP1A1 generated the smallest amount of ROS (3.9 pmol/min), with CYP1B1 being intermediate (6.1 pmol/min) and CYP1A2 producing the most (11.1 pmol/min). The effects of combinations of particles and CYP1s are described below.

Beginning with CYP1A1 (Figure 3A), the addition of CYP1A1 to the CuO-Si group caused a synergistic increase in ROS generation when compared to the particles alone. A similar degree of synergistic stimulation of ROS generation was also observed with MCP230. Based on the slopes, both CuO-Si and MCP230 produced a similar degree of ROS stimulation (56.6 and 51.1 pmol/min, respectively).

A similar response was observed with CYP1A2 (Figure 3B). An increase in the CYP1A2 concentration for both the EPFR and non-EPFR particles produced an increase in ROS that appeared to plateau at CYP1A2 concentrations above 0.08 μM. When comparing the slopes of the lines (using the linear response range), the results are consistent with CYP1A2, leading to a synergistic stimulation of the particle-mediated ROS production. Again, both CuO-Si and MCP230 produced a similar degree of stimulation (67.2 and 79.6 pmol/min, respectively), although both were significantly greater than with CYP1A2 alone.

CYP1B1 (Figure 3C) produced a response similar to the CYP1As. In the presence of either set of particles, an increase in CYP1B1 caused a linear increase in ROS production that was greater than that of CYP1B1 in the absence of particles. The slopes in the presence of CuO-Si and MCP230 were 55.1 and 52.9 pmol/min, respectively, and clearly showed that ROS generation was synergistically stimulated in the presence of CYP1B1.

This raised an important question with regard to what was driving this stimulation. Although both CYP1A1 and CYP1A2 retain intact POR•CYP1 complexes in the presence of the particles, CYP1B1 differs in that the POR•CYP1B1 BRET complex was readily disrupted by MCP230, but not CuO-Si. Despite these differences, (1) each of the CYP1s generated similar amounts of ROS, and (2) both EPFR and non-EPFR particles produced similar degrees of synergistic stimulation with CYP1B1 despite not having an intact POR•CYP1B1 complex. In order to address this inconsistency, ROS generation was examined for each of the P450s using both intact and partial reconstituted systems.

**Figure 4:**
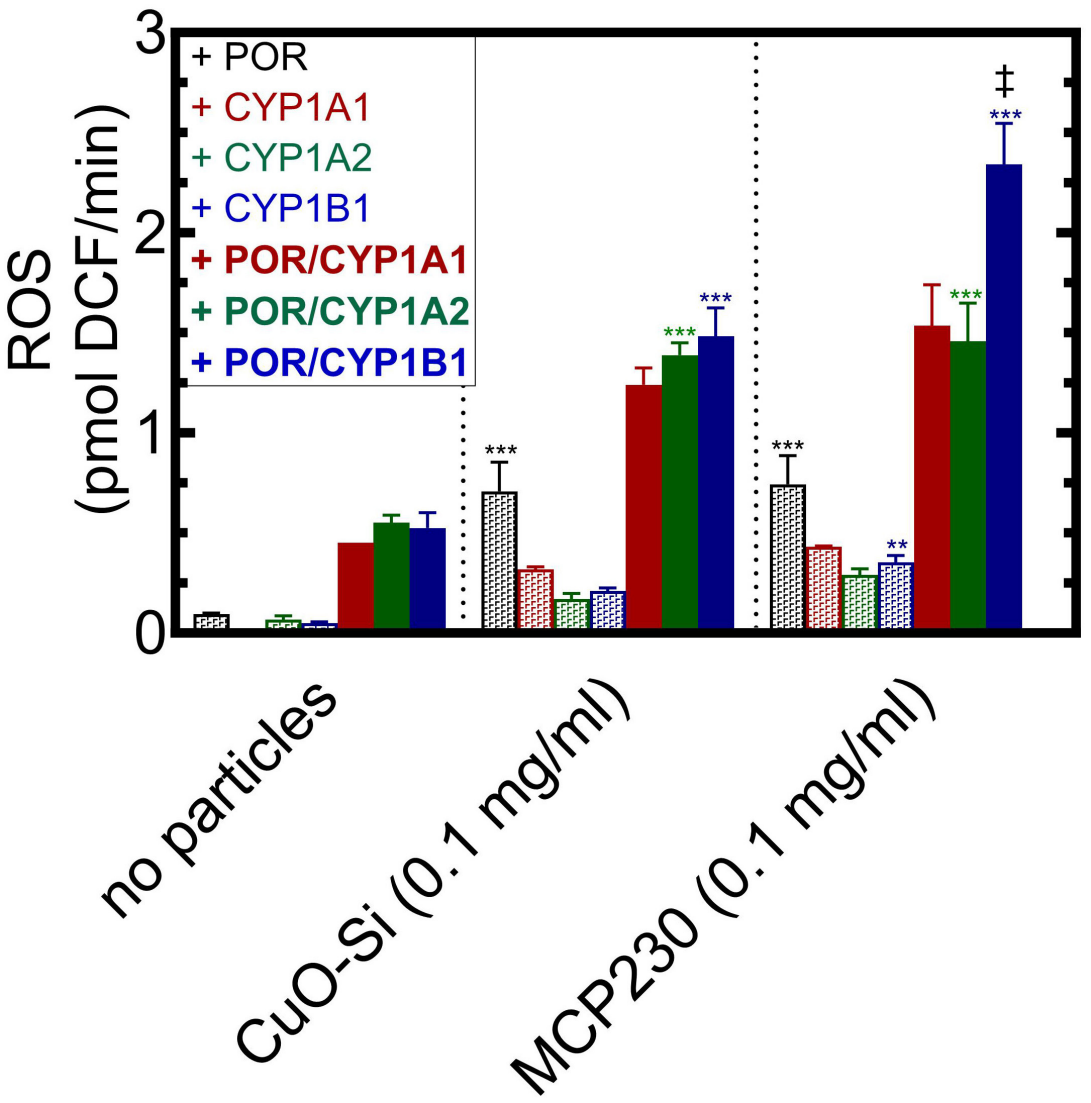
Identification of the components of the CYP1-containing reconstituted systems responsible for synergistic ROS generation. ROS formation was measured in the absence and presence of particulate matter when combined with components of a CYP1-containing reconstituted system. The Figure is divided into three sections, no particles **(left)**, the non-EPFR, CuO-Si **(center)**, and the EPFR, MCP230. In each case, ROS generation was measured in the presence of POR (black), CYP1A1 (red), CYP1A2 (green), CYP1B1 (blue). The hatched bars represent partial reconstituted systems using only the proteins indicated. The solid bars represent complete reconstituted systems that contain both POR and the indicated CYP1 enzyme. Data are shown as the mean ± SD. Because of lack of protein availability, there is only a single determination for CYP1A1 in the absence of particles, and duplicate determinations for CYP1A1 in the CuO-Si and MCP230 groups. *N* = 4 for all other conditions. Because of the low *N* for the CYP1A1 data, it was not included in the statistical analysis. Statistics were performed using a one-way analysis of variance and Bonferroni's multiple comparison test (significantly different from the corresponding group in the absence of particles – **, *p* < 0.01; and ***, *p* < 0.001). ‡ – significantly different from the corresponding CuO-Si group (*p* < 0.01). In all cases, a synergistic stimulation of ROS production was observed when comparing the complete reconstituted system (POR•CYP1) vs. the sum of POR and CYP1 alone (*p* < 0.01, not shown for clarity).

In Figure 4, stimulation of ROS generation was examined in the presence of complete and partial reconstituted systems with each of the particle systems. First, in the absence of particles (left), the incomplete reconstituted system containing only POR (black hatched bar) showed virtually no ability to generate ROS. The same could be said for the partial systems that contained only the P450 enzymes. However, when the complete reconstituted systems containing both POR and the CYP1 enzymes were present, a moderate amount of ROS was generated (solid bars, no particles).

In the presence of CuO-Si (Figure 4, center), POR alone was able to generate a moderate amount of ROS (black hatched bars), which suggests that electron transfer to POR can participate in free radical production when particles are present. Interestingly, each of the partial systems containing only the P450s was also able to interact with CuO-Si to generate ROS. When the complete reconstituted systems were examined, ROS production was further stimulated.

A similar but greater response was observed in the presence of the EPFR, MCP230, particularly with CYP1B1 (Figure 4, right). Again, POR alone was able to interact with the EPFR-containing particle to produce a similar amount of ROS (black hatched bars) as was observed with the non-EPFR CuO-Si. A smaller amount of ROS generation was also observed when the systems containing only the P450s and MCP230 were combined, again similar to that observed with CuO-Si. Combination of the complete reconstituted systems with MCP230 caused an increase in ROS that was greater than the partial systems (POR or the CYP1s alone).

Interestingly, the MCP230-POR•CYP1B1 system (Figure 4, right, solid blue bars) was stimulated to a greater extent than was observed with MCP230-POR•CYP1A1 or MCP230-POR•CYP1A2. When comparing the non-EPFR (center) vs. EPFR (right) particles, the stimulation of CYP1B1-mediated ROS in the EPFR-containing group was much greater than seen with CuO-Si. The reasons for these differences will require further study.

It is important to note that, when in the presence of particles, ROS stimulation of the complete POR•CYP1 reconstituted systems were synergistic. That is, the stimulation by the complete systems (solid bars) were greater than that of POR alone (black hatched bars) plus CYP1 alone (colored hatched bars).

## Discussion

Members of the CYP1 family are known to possess significant sequence similarity. Human CYP1A1 and CYP1A2 share 72% sequence identity, whereas their sequence identity to CYP1B1 is about 40% (40). Despite their similarities in sequence, these forms exhibit different functional characteristics. For example, CYP1A1 and CYP1A2 form homomeric complexes, whereas CYP1B1 does not (41)—only CYP1A1 and CYP1B1 form heteromeric complexes with other enzymes of the CYP1 family. CYP1A2 and CYP1B1 both exist predominantly in the ordered lipid raft regions of the endoplasmic reticulum, with CYP1A1 preferring the disordered membrane regions (41). With the potential of each of these forms to generate reactive oxygen as well as their potential to deactivate endogenous AhR agonists such as FICZ, we were interested in determining whether the CYP1 enzymes differed with respect to their response to the presence of EPFRs.

As was seen with several other P450s, EPFRs caused a generalized inhibition of monooxygenase activity (12, 13, 24, 25). Both CuO-Si and MCP230 were moderate inhibitors of CYP1A1-mediated 7BR metabolism, with MCP230 being slightly more effective. Both particles were more effective inhibitors of CYP1A2-mediated 7BR metabolism than seen with CYP1A1; however, with both CYP1A proteins, there was no significant difference between the EPFR and non-EPFR particles. In contrast, CYP1B1 exhibited different behavior being inhibited by both particles to a much greater extent. Surprisingly, in this study, the non-EPFR CuO-Si was a more effective inhibitor than MCP230 (Figure 1C). These results differ from those observed with rabbit CYP2B4 (13), rabbit CYP1A2 (24), as well as several rat P450s (12) where the EPFR was a more effective inhibitor than the non-EPFR particles.

When examining complex formation, we observed substantial differences among the P450s. Whereas complex formation between POR and either CYP1A1 or CYP1A2 was not affected by either particle species, the POR•CYP1B1 complex was significantly disrupted in the presence of MCP230, but not the non-EPFR, CuO-Si. Although disruption of this redox complex would clearly be expected to inhibit monooxygenase activity (Figure 1C), it does not explain why a similar degree of inhibition was observed with the non-EPFR particle. Understanding this discrepancy will require further study.

The effects of EPFRs on CYP1-mediated ROS generation were also interesting. As shown in Figure 3, the reconstituted systems containing POR and CYP1 led to a synergistic stimulation of ROS generation that was observed for both the MCP230 and CuO-Si groups. POR alone (which can provide electrons) stimulated ROS production in the presence of particulate matter; however, each of the P450 enzymes alone was also able to generate ROS, although at lower levels. Interestingly, the complete reconstituted systems, containing both POR and P450, produced a greater amount of ROS than any of the partial systems. Taken together, these results indicate that P450-dependent monooxygenase activity is significantly inhibited by both MCP230 and CuO-Si and that despite this inhibition, ROS generation is synergistically stimulated. Both POR and the CYP1 enzymes alone were capable of interacting with particulate matter to generate ROS: the complete reconstituted systems led to a greater degree of stimulation.

The overall inhibition of monooxygenase activity can be particularly important when considering the Ah receptor response. Numerous compounds are known to be AhR activators. Some interact directly with AhR, whereas others act indirectly through the generation of ROS (42). Under conditions where ROS is generated, the radical species can convert tryptophan to the known endogenous AhR agonist, FICZ. This will lead to the typical AhR response of respiratory, cardiovascular, and immune effects, as well as induction of CYP1 proteins. In the presence of AhR activation, which is mediated by the conversion of tryptophan to the endogenous FICZ, the FICZ can be inactivated by CYP1-mediated metabolism, which terminates the response.

EPFRs are known to cause detriments to the respiratory and cardiovascular systems, changes in immune response, and in CYP1 expression by activation of the Ah receptor, most likely due to the generation of ROS. However, EPFRs, and potentially other particulates, may affect the AhR response in multiple ways, first by their propensity to generate ROS and second by their ability to inhibit CYP1 monooxygenase activity. Our data now shows that both MCP230 and CuO-Si interact with P450s to synergistically stimulate ROS generation, which can lead to increased conversion of tryptophan to FICZ. This ROS stimulation is independent of the presence of an intact reconstituted system (containing both POR and P450), but some ROS can be generated in the presence of either POR or P450 alone. This means that there is the potential for all the P450s in a cell to generate ROS in the presence of particulate matter, and not just those that are in complex with POR.

The second feature of the EPFR response is related to its ability to inhibit CYP1 monooxygenase activity. Inhibition of CYP1 monooxygenase activities will not only inhibit the metabolism and clearance of drugs and foreign compounds, but can also prolong AhR activation. FICZ is inactivated by CYP1A1 and possibly the other CYP1 proteins. EPFRs inhibit CYP1A, which would inhibit the inactivation of FICZ, prolonging the AhR response.

Extrapolation of these *in vitro* results to the *in vivo* situation is difficult to predict at this time. However, each of the P450s examined so far have shown inhibition of monooxygenase function and synergistic stimulation of ROS generation, which allows us to suggest that similar effects will be found *in vivo*. Further study will be required to uncover these details.

In summary, particulate matter causes a significant inhibition of CYP1-dependent monooxygenase activities. The EPFR leads to the specific disruption of POR complex formation with CYP1B1 but not with those from the CYP1A subfamily. Finally, particle-mediated ROS generation is synergistically stimulated when in the presence of POR•CYP1 reconstituted systems, although an interaction between the P450 and its redox partner, POR, is not required for at least some of the ROS production. Further study will be required to identify the components of the particulate matter responsible for these effects, be it the metal or the particulate matter itself.

## Data Availability

The original contributions presented in the study are included in the article/supplementary material, further inquiries can be directed to the corresponding author.
